# Threshold-Independent Amide Proton Transfer-Weighted MRI in Post-Treatment Gliomas: A Systematic Review and Meta-Analysis of Effect Size and Diagnostic Accuracy

**DOI:** 10.3390/life16101584

**Published:** 2026-09-22

**Authors:** Gianvincenzo Sparacia, Alberto Iaia, Giuseppe Mamone, Roberto Miraglia

**Affiliations:** 1 Department of Radiology, Mediterranean Institute for Transplantation and Advanced Specialized Therapies (IRCCS ISMETT), 90127 Palermo, Italy; gmamone@ismett.edu; 2 Department of Radiology, University of Pittsburgh Medical Center (UPMC) Italy, 90127 Palermo, Italy; rmiraglia@ismett.edu; 3 Department of Biomedicine, Neuroscience and Advanced Diagnostics (BiND), Università Degli Studi di Palermo, 90127 Palermo, Italy; 4 Department of Neuroradiology, Christiana Care Health System, Wilmington, DE 19801, USA; iaia26@comcast.net

**Keywords:** amide proton transfer-weighted MRI, chemical exchange saturation transfer, glioma, pseudoprogression, treatment-related effects, progressive disease, systematic review, meta-analysis, Hedges’ *g*

## Abstract

Post-treatment glioma surveillance often cannot reliably separate progressive disease (PD) from treatment-related effects (TRE) using conventional MRI alone. Amide proton transfer-weighted (APTw) MRI, a chemical exchange saturation transfer technique that reflects tissue protein content, has been investigated for this purpose; however, existing meta-analyses have quantified only cutoff-dependent diagnostic accuracy, and the magnitude of APTw signal separation has not been systematically synthesized. A systematic literature search was performed in PubMed and Web of Science (7 July 2026; no language restrictions). The same search strategy was applied to both databases. The search identified seven eligible studies. Five studies reporting continuous data (n = 218 patients) were pooled for the standardized mean difference (Hedges’ *g*) in APTw signal between PD and TRE. The pooled Hedges’ *g* was 1.54 (95% CI, 0.73–2.35; *p* = 0.006, Hartung–Knapp adjustment). Between-study heterogeneity was 63.9%, and the 95% prediction interval ranged from 0.04 to 3.04. Six studies provided diagnostic accuracy data, yielding pooled sensitivity, specificity, and HSROC AUC values of 0.88, 0.78, and 0.86, respectively. Case definitions varied across studies: four studies confirmed 19–40% of cases histopathologically, whereas three relied exclusively on radiologic follow-up. The limited number of available studies precluded subgroup analyses and molecular stratification. The small number of included studies and the high between-study heterogeneity (I^2^ = 63.9%) limit the generalizability of these results. All five studies reported higher APTw signal in PD than in TRE; however, the magnitude of this difference remains imprecisely estimated and requires confirmation in prospective, homogeneous cohorts.

## 1. Introduction

Among primary malignant brain tumors, high-grade gliomas remain associated with poor outcomes despite standard-of-care management, consisting of maximal safe surgical resection followed by concurrent radiotherapy and adjuvant temozolomide-based chemoradiotherapy [1]. On follow-up MRI, new or enlarging contrast-enhancing lesions appear in up to 30% of patients and may reflect either true tumor regrowth (progressive disease, PD) requiring second-line therapy, or treatment-related effects (TRE), encompassing pseudoprogression and radiation necrosis, managed conservatively [2,3]. Misclassification carries direct consequences: patients with PD mistakenly managed as TRE may lose the opportunity for timely second-line treatment, while those with TRE unnecessarily treated as PD are exposed to toxic agents [4].

Standard MRI sequences are sensitive to edema and disruption of the blood–brain barrier, but these findings do not discriminate PD from TRE, since both entities can produce similar enhancement, surrounding edema, and mass effect [5]. Histopathology remains the definitive reference standard, but repeat biopsy is invasive and intratumoral heterogeneity can produce false-negative results [6,7]. A noninvasive method able to resolve this distinction at a single imaging time point is therefore needed.

Amide proton transfer-weighted (APTw) MRI belongs to the family of chemical exchange saturation transfer (CEST) techniques, deriving contrast from the transfer of magnetization between exchangeable amide protons on endogenous mobile proteins and peptides and the surrounding bulk water, without any need for exogenous contrast material; the resulting effect is captured through magnetization transfer ratio asymmetry measured at 3.5 ppm, or MTRasym (3.5 ppm) [8,9]. The biological rationale rests on differences in protein content and cell density: viable, proliferating tumor tissue is rich in cytosolic protein and densely cellular, producing a higher APTw signal, whereas TRE tissue undergoes coagulative necrosis with loss of cellular architecture, yielding a lower or near-isointense signal [10,11]. Preclinical models and, subsequently, image-guided stereotactic biopsy studies have confirmed this pattern [12,13]. Primary studies have reported a higher APTw signal in PD than in TRE, with AUC values ranging from 0.77 to 0.98 [14,15,16,17,18,19,20,21]. This variability reflects differences in cohort size, glioma grade, acquisition technique, ROI definition, and reference standard, limiting direct comparisons across studies. Three prior meta-analyses of APTw MRI in this setting have all pooled diagnostic accuracy: Essed et al. [22] reported pooled sensitivity of 0.88 and specificity of 0.84; and Chen et al. [23] and Taylor et al. [24] found high but heterogeneous estimates. Sensitivity and specificity are cutoff-dependent: each study selects its own optimal MTRasym (3.5 ppm) threshold, so pooled accuracy reflects both the true signal separation and the threshold chosen, not signal separation alone. None of these syntheses quantifies how large the APTw difference between PD and TRE is, or whether it is preserved across acquisition protocols and clinical settings. No meta-analysis has addressed this on a threshold-independent scale. Hedges’ *g* quantifies the between-group difference in standard deviation units, independently of any diagnostic cutoff; its prediction interval captures the variability of this effect across settings.

We aimed to quantify the threshold-independent separation in APTw signal between PD and TRE in post-treatment gliomas, expressed as pooled Hedges’ *g*. Secondary objectives were to summarize diagnostic accuracy by HSROC AUC, derive exploratory weighted mean APTw signal values for each group, assess between-study heterogeneity, and appraise study quality.

## 2. Materials and Methods

### 2.1. Search Strategy and Study Selection

This systematic review and meta-analysis followed PRISMA 2020 guidelines [25]; the completed checklist is provided in the Supplementary Materials. No prospective registration was undertaken (see Limitations). PubMed was searched using Medical Subject Headings combined with free-text terms; a confirmatory search was run in the Web of Science Core Collection using an equivalent topic-search string: TS = (“amide proton transfer” OR “amide proton transfer-weighted” OR APT OR APTw OR “chemical exchange saturation transfer” OR CEST) AND TS = (glioma* OR glioblastoma* OR astrocytoma* OR oligodendroglioma* OR “brain neoplasm*”). Embase and Scopus were not searched owing to lack of institutional access; this is noted as a limitation. An initial search was performed in April 2026 and updated on July 7, 2026, with no language restrictions, using the following PubMed string: (“amide proton transfer”[tiab] OR “amide proton transfer-weighted”[tiab] OR APT[tiab] OR APTw[tiab] OR “chemical exchange saturation transfer”[tiab] OR CEST[tiab]) AND (“Glioma”[Mesh] OR glioma*[tiab] OR glioblastoma*[tiab] OR astrocytoma*[tiab] OR oligodendroglioma*[tiab] OR “Brain Neoplasms”[Mesh]). The PubMed search returned 372 records, and the Web of Science search (10 July 2026) returned 325 records, with no additional eligible studies identified after cross-checking against the PubMed set.

Eligibility for the review required: (1) adult patients with a histopathologically confirmed glioma of any WHO grade; (2) prior treatment with radiotherapy or chemoradiotherapy; (3) APTw MRI acquired at 3 Tesla with MTRasym (3.5 ppm); and (4) a final PD or TRE diagnosis established either histopathologically or through at least 6 months of longitudinal follow-up according to the Response Assessment in Neuro-Oncology criteria. Inclusion in the primary continuous-outcome synthesis further required that a study report, or allow derivation of, the mean and standard deviation (SD) of the APTw signal for PD and TRE separately within contrast-enhancing regions of interest (ROIs). Studies unable to meet this requirement were excluded from the standardized mean difference (SMD) analysis but could still contribute to the diagnostic test accuracy (DTA) synthesis provided that sensitivity, specificity, and sample size were reported. Studies of brain metastases or animal models, and any study lacking data extractable for either synthesis, were excluded.

### 2.2. Data Extraction

Two reviewers independently extracted the study characteristics, sample size, APTw metric, acquisition type, reference standard, and mean and SD of APTw signal in contrast-enhancing ROIs for PD and TRE when available. The reference standard used to confirm PD or TRE (histopathological confirmation versus radiologic follow-up) was recorded for each study, together with the specific response-assessment criteria applied where reported: the original RANO criteria [2] for Ma et al. [14] and RANO 2.0 for Karimian-Jazi et al. [20] and Zeyen et al. [21]; details are provided in Table 1.

The mean APTw value in the contrast-enhancing ROI was prespecified as the primary SMD metric. When SDs were not reported, they were estimated by back-calculation from the two-sample *t*-test statistic. No values were imputed from graphical data. Disagreements were resolved by consensus.

### 2.3. Continuous Outcome Meta-Analysis (Standardized Mean Difference)

The primary outcome was the SMD in APTw signal intensity between PD and TRE, calculated as Hedges’ *g* with small-sample bias correction [26]. A random-effects model with restricted maximum likelihood estimation was used for pooling [27], given expected heterogeneity in glioma grade, treatment history, ROI strategy, and acquisition parameters. Confidence intervals for the pooled effect were computed with the Hartung–Knapp adjustment, and a 95% prediction interval was calculated to describe the range of true effects expected across settings. Exploratory weighted mean APTw signal values for PD and TRE were derived as inverse-variance weighted means across studies with extractable continuous data on a positive signal scale.

### 2.4. Diagnostic Test Accuracy (Secondary Outcome)

Contingency (2 × 2) tables were reconstructed from the reported sensitivity, specificity, and sample size using standard rounding: true positives (TP) = round(n_PD × sensitivity), false negatives (FN) = n_PD − TP, true negatives (TN) = round(n_TRE × specificity), and false positives (FP) = n_TRE − TN, where n_PD and n_TRE are the reported group sample sizes. Two complementary syntheses of diagnostic accuracy were performed. First, sensitivity and specificity were pooled separately using univariate random-effects models with logit transformation [28]; these estimates (sensitivity, 0.88; specificity, 0.78) are reported as the primary diagnostic accuracy summary because they retain the most direct correspondence with each study’s original 2 × 2 data. Second, a bivariate Reitsma model was fitted to jointly estimate sensitivity and specificity while accounting for their within-study correlation, yielding the HSROC curve, its AUC, and a summary operating point (sensitivity, 0.83; specificity, 0.75); this bivariate point is a model-based projection onto the HSROC curve rather than a direct average of the univariate estimates, and the two sets of values are expected to differ modestly for this reason. Karimian-Jazi et al. [20] were excluded from both analyses because sensitivity and specificity were not reported numerically. All analyses were performed in R (Version 4.5.3, R Foundation for Statistical Computing, Vienna, Austria) using the meta, metafor, and mada packages [27,29].

### 2.5. Heterogeneity and Publication Bias

Statistical heterogeneity was assessed using *I*^2^, Cochran’s *Q*, and *τ*^2^, each reported with corresponding 95% confidence intervals [30]. Egger’s test for funnel plot asymmetry was omitted because fewer than 10 studies contributed to the primary synthesis, a threshold below which regression-based tests lack statistical power and yield inflated false-positive rates [31]; the funnel plot was evaluated via visual inspection only. Sensitivity and robustness were evaluated using sequential leave-one-out cross-validation across SMD studies.

In accordance with the Cochrane Handbook and PRISMA guidelines, formal meta-regression and subgroup analyses (e.g., by WHO grade, 2D vs. 3D acquisition schemes, or molecular markers including IDH mutation, MGMT promoter methylation, and 1p/19q codeletion) require a minimum of 10 studies per candidate moderator to avoid overfitting and type I errors. Given the restricted number of eligible studies (*k* = 5 for SMD and *k* = 6 for DTA), quantitative subgroup explorations were prespecified as non-feasible and were instead systematically addressed through narrative synthesis in the Discussion and Limitations sections.

A formal certainty of evidence assessment (e.g., the GRADE framework) was not applied, as standardized GRADE protocols for continuous imaging effect sizes with heterogeneous, follow-up-based reference standards remain unestablished.

### 2.6. Risk-of-Bias Assessment

Methodological quality was assessed with the Quality Assessment of Diagnostic Accuracy Studies 2 (QUADAS-2) tool [32] across patient selection, index test, reference standard, and flow and timing. Two reviewers rated each domain as having a low, high, or unclear risk of bias; disagreements were resolved by consensus.

## 3. Results

### 3.1. Study Selection and Characteristics

The search identified 372 records from PubMed and 325 records from Web of Science. After removal of 309 duplicate records, 388 unique records underwent title and abstract screening. Of these, 350 were excluded, and 38 full-text articles were assessed for eligibility. Thirty-one full-text articles were excluded for the reasons summarized in Figure 1, leaving seven studies that met the inclusion criteria. Screening of the reference lists identified no additional eligible studies.

Table 1 summarizes the characteristics of the included studies. All studies used MTRasym (3.5 ppm) at 3 Tesla; sample sizes ranged from 30 to 64 patients. Acquisition was 3D in five studies and 2D in two. Examples of excluded studies are listed in the Supplementary Materials. Five studies contributed to the primary SMD analysis (Ma et al. [14], Chen et al. [19], Karimian-Jazi et al. [20], Zeyen et al. [21], and Liu et al. [15]), and six to the diagnostic accuracy analysis. Park et al. [16] and Hou et al. [17] contributed to the accuracy analysis only because group means were not numerically reported; Karimian-Jazi et al. [20] contributed to the SMD analysis only.

### 3.2. Standardized Mean Difference of APTw Signal

All five studies reported a higher APTw signal in PD than in TRE; individual SMDs ranged from 0.95 to 2.76. The pooled Hedges’ *g* was 1.54 (95% CI, 0.73–2.35; *p* = 0.006, Hartung–Knapp adjustment; Figure 2). Heterogeneity estimates were I^2^ = 63.9% (95% CI, 0.0–96.7%), Q = 9.93 (df = 4; *p* = 0.04), and τ^2^ = 0.235. The 95% prediction interval ranged from 0.04 to 3.04. In the leave-one-out analysis, Hedges’ *g* ranged from 1.33 after exclusion of Ma et al. [14] to 1.69 after exclusion of Karimian-Jazi et al. [20].

The weighted mean APTw signal was 2.85% (SD, 0.40) for PD and 1.75% (SD, 0.61) for TRE, based on MTRasym at 3.5 ppm in the four studies reporting extractable continuous data on a positive signal scale. Studies using a negative MTRasym scale (Liu et al. [15]), APTw values normalized to white matter (Hou et al. [17]), or data without an extractable standard deviation (Park et al. [16]) were excluded from this analysis. These estimates are therefore specific to the positive MTRasym scale and cannot be compared directly with values obtained using other signal scales. Optimal cutoffs reported in three studies ranged from 1.97% to 2.75%.

### 3.3. Diagnostic Test Accuracy (Secondary Outcome)

Across six studies, pooled sensitivity was 0.88 (95% CI, 0.79–0.93; Figure 3A) and pooled specificity was 0.78 (95% CI, 0.69–0.85; Figure 3B). HSROC AUC was 0.86, with a summary operating point of 0.83 sensitivity and 0.75 specificity (Figure 4). Chen et al. [19] showed the highest sensitivity (1.00), whereas Zeyen et al. [21] showed the lowest (0.75), consistent with the broader temporal distribution of its clinical-practice cohort. All meta-analytic results are summarized in Table 2.

### 3.4. Publication Bias

The funnel plot for the five SMD studies is shown in Figure 5. Visual inspection suggested possible asymmetry, with Ma et al. [14] showing a high effect estimate and a high standard error. Egger’s test was not performed because fewer than 10 studies were available [31]. The funnel plot was therefore considered exploratory.

### 3.5. Risk-of-Bias Assessment

QUADAS-2 results are summarized in Figure 6. Risk of bias was low across most domains. Park et al. [16] was rated as being at high risk of bias in the index test domain because APTw readers were not blinded to the clinical outcome. Chen et al. [19] was rated as being at high risk of bias in the Patient Selection domain and had high applicability concern in the Reference Standard domain. All seven studies were rated as being at unclear risk of bias in the Reference Standard domain because none explicitly reported whether the final classification of PD or TRE was made without knowledge of the APTw results. No study was rated as being at high risk of bias in the Reference Standard or Flow and Timing domains.

## 4. Discussion

In all seven included studies, APTw signal was higher in PD than in TRE, independently of any diagnostic threshold. The pooled Hedges’ *g* was 1.54, with a 95% prediction interval of 0.04–3.04; derived from five studies only, this interval does not exclude a negligible effect in some clinical settings. This pattern aligns with the diagnostic accuracy findings (sensitivity 0.88, specificity 0.78, HSROC AUC 0.86) and fits a plausible biological explanation: viable tumor tends to have higher cytosolic protein content and cell density, while treatment-related tissue is largely necrotic, with disrupted cellular architecture [10,11].

Standardization permits the combination of results reported on different scales, but differences in acquisition protocols, ROI definitions, and signal processing may still affect the estimated effect sizes.

A methodological concern specific to this literature deserves emphasis. Three of the five studies contributing to the primary Hedges’ *g* (Ma et al. [14], Karimian-Jazi et al. [20], and Zeyen et al. [21]) defined PD and TRE primarily by radiologic follow-up trajectory according to RANO criteria, whereas the remaining two (Chen et al. [19] and Liu et al. [15]) relied on histopathological confirmation in only 39% and 23% of cases, respectively, with follow-up imaging used for the remainder (Table 1). Because APTw MRI is itself an imaging biomarker, and imaging-based follow-up was used as part of the reference standard in several studies, some degree of circularity cannot be excluded. A lesion ultimately classified as TRE, on the basis of radiological stability, may be more likely to exhibit a lower baseline APTw signal, potentially inflating the apparent association between imaging findings and final classification. The QUADAS-2 assessment identified methodological concerns in two of the seven included studies, including high risk of bias in the index test domain for Park et al. and high risk of bias in Patient Selection together with a Reference Standard applicability concern for Chen et al. These findings highlight limitations in the underlying evidence base rather than in the meta-analytic methodology itself.

The Reference Standard domain in QUADAS-2 considers whether the reference standard is likely to classify the target condition correctly and whether its interpretation was performed without knowledge of the index test result. The original studies were therefore reassessed for explicit reporting of blinding to the APTw findings. When blinding was not reported, the Reference Standard domain was rated as being of unclear risk of bias. The applicability concern for Chen et al. reflects the reference standard used to define the target condition and was assessed separately from the risk-of-bias judgment.

Three earlier meta-analyses pooled the diagnostic accuracy of APTw MRI in this setting [22,23,24]. The present HSROC area under the curve of 0.86 was close to, though lower than, the 0.93 reported by Essed et al. [22]. This likely reflects the present restriction to studies with extractable continuous APTw values and the inclusion of recent, clinically heterogeneous cohorts (Zeyen et al. [21]; Karimian-Jazi et al. [20]) with wider variation in timing, treatment history, and glioma grade than earlier, more selected series. The threshold-independent effect size adds information that pooled accuracy alone cannot provide: the pooled Hedges’ *g* of 1.54 is comparable to, or greater than, effect sizes reported for diffusion- and perfusion-weighted MRI in the same setting [24], and, unlike sensitivity and specificity, it does not depend on the cutoff each study selected.

Reported optimal cutoffs ranged from 1.97% to 2.75% across the three studies providing this information. These absolute values are not transferable across signal scales or acquisition protocols and should not be interpreted as validated clinical thresholds; the threshold-independent effect size, rather than any single cutoff, remains the primary summary of the between-group difference reported in this review.

The pooled SMD of 1.54 was lower than estimates from some single-center studies, likely reflecting broader clinical heterogeneity, treatment timing, and patient selection. Leave-one-out analysis supported the numerical stability of the point estimate, but the imprecisely estimated heterogeneity (I^2^ = 63.9%; 95% CI, 0.0–96.7%) indicates that APTw performance is not uniform across clinical settings. This heterogeneity is unlikely to be explained by cutoff selection alone, since Hedges’ *g* is threshold-independent; more plausible sources include the span of WHO grades (II–IV) represented across the included cohorts, the mix of 2D and 3D acquisitions, and variation in time from radiotherapy to imaging. Molecular stratification by IDH mutation status, MGMT promoter methylation, or 1p/19q codeletion, all of which are known to affect both glioma behavior and treatment response, was not reported with sufficient granularity in the primary studies to allow subgroup analysis, and its absence limits the clinical specificity of the pooled estimate.

Post-treatment timing may partly explain this heterogeneity. Zeyen et al. [21] reported time-dependent diagnostic performance in IDH-wildtype glioblastoma: AUC was 0.93 within 6 months after radiotherapy, 0.76 at later follow-up, and 0.67 at second progression.

These findings suggest that the diagnostic performance of APTw MRI may vary according to the interval from radiotherapy, but confirmation in additional cohorts is required.

Anti-angiogenic therapy may also affect APTw interpretation. Zeyen et al. [21] reported lower APTw signal in PD after bevacizumab administered within 1 month before imaging (median, 1.53% versus 2.36% without bevacizumab; *p* = 0.01), potentially increasing false-negative assessments. This effect was not systematically addressed across studies and may be relevant in later-stage disease.

A further interpretive pitfall concerns the potential confounding of the APTw signal by blood products and coagulative necrosis, both common within post-treatment glioma cavities and not explicitly addressed in the primary studies. Blood degradation products substantially elevate APTw signal: in hemorrhagic brain lesions, magnetization transfer ratio asymmetry at 3.5 ppm has been reported as 3.68% in acute hemorrhage and 1.6% in subacute hemorrhage, values that exceed and approach, respectively, the pooled weighted mean APTw signal observed here for PD (2.85%) and TRE (1.75%) [33]. Because postoperative and postradiation cavities frequently contain residual or recurrent hemorrhage, an enhancing focus containing blood products could be misclassified as PD, on the basis of an elevated APTw signal that in fact reflects hemoglobin degradation rather than viable tumor. Coagulative necrosis, the histological hallmark of TRE, can itself be heterogeneous, and areas of liquefactive or cystic change within a necrotic focus may further lower the APTw signal independently of tumor viability, potentially widening the apparent separation between PD and TRE for reasons unrelated to disease biology. None of the included studies reported explicit ROI exclusion or correction for hemorrhagic or necrotic-fluid components; this represents a plausible source of both false-positive and false-negative APTw classifications that future studies should address through careful ROI curation or fluid-suppressed acquisition strategies.

Methodological variability remains an important limitation. Differences in saturation schemes, ROI placement, and signal normalization may affect absolute APTw values. The standardized acquisition protocols recommended by Zhou et al. [34] are needed to improve reproducibility across institutions and MRI systems.

## 5. Limitations

Several limitations inherent to the available evidence base must be considered when interpreting these findings.

First, the primary synthesis relies on a small number of eligible cohorts (*k* = 5 for SMD and *k* = 6 for DTA). While the 95% prediction interval (0.04 to 3.04) narrowly excludes zero, its wide range reflects substantial variability across settings. The precise magnitude of signal separation remains uncertain in specific clinical environments.

Second, the reference standard used to differentiate PD from TRE varied markedly across primary studies, ranging from radiologic follow-up trajectory according to RANO criteria to histopathological verification in only 19–40% of cases. Relying on longitudinal imaging follow-up as ground truth introduces structural reference standard circularity, as APTw MRI is itself an imaging biomarker. This constraint stems from the primary literature rather than meta-analytic design. Noninvasive estimates should be interpreted as measures of clinical association under imperfect ground-truth conditions rather than definitive diagnostic accuracy. In addition, the primary studies did not consistently operationalize the TRE category in the same way: three defined their comparator group specifically as pseudoprogression [14,20,21], one as radiation-induced brain injury [19], and three used broader, non-specific terms such as treatment-induced change or treatment response [15,16,17] without separately reporting pseudoprogression and radiation necrosis as distinct subgroups. Because these two entities differ biologically and typically occur at different intervals after radiotherapy, this terminological heterogeneity represents an additional, unquantifiable source of clinical heterogeneity that could not be addressed through subgroup analysis given the small number of studies.

Third, included cohorts spanned WHO grade II–IV gliomas, 2D and 3D acquisition schemes, and varying intervals post-radiotherapy. Primary studies lacked sufficient data on key molecular markers (IDH mutation status, MGMT promoter methylation, and 1p/19q codeletion) to perform formal subgroup analyses or meta-regression. The pooled estimate reflects an overall average across a heterogeneous patient population rather than a molecularly stratified value.

Fourth, PubMed was supplemented by a search of the Web of Science Core Collection, which identified 325 records but no additional eligible studies after duplicate removal and screening. Embase and Scopus were not searched because institutional access to these databases was not available to any of the authors; relevant studies indexed exclusively in these databases may therefore have been missed.

Fifth, this review was not prospectively registered. Prospective registration requires submission before the review is undertaken; in this case, the decision to formalize the work as a fully registered systematic review was made only after the literature had already been assembled and an initial synthesis performed, precluding retrospective registration. Although the primary outcome (Hedges’ *g* for APTw signal separation) and the overall analytic approach were fixed before formal statistical pooling, no written protocol prespecifying the complete analysis plan was established beforehand; the absence of prospective registration limits the ability to fully distinguish prespecified from post hoc analytic choices, and this should be considered when interpreting the results.

Finally, standard deviations were backcalculated for select studies where direct continuous parameters were not reported.

## 6. Future Directions

Future studies should use standardized APTw acquisition protocols, adopt histopathological confirmation as the reference standard where feasible to reduce circularity, and stratify patients by time from radiotherapy and concomitant treatment. Correlation with molecular markers, including IDH mutation status and MGMT promoter methylation, may help define subgroups in which APTw MRI is most useful. Because several included studies suggest that multimodal imaging outperforms APTw alone [16,17,19,20], the incremental value of APTw MRI should be tested prospectively against diffusion- and perfusion-weighted imaging. An updated systematic review incorporating additional databases (e.g., Embase, Scopus) and individual patient data, once a sufficient number of studies accumulate, would allow for formal subgroup and meta-regression analyses that are not currently feasible.

## 7. Conclusions

In this systematic review and meta-analysis, APTw signal was higher in PD than in TRE in all included studies, with a pooled Hedges’ *g* of 1.54, supported by moderate-to-high diagnostic accuracy (HSROC AUC, 0.86; pooled sensitivity, 0.88; pooled specificity, 0.78). However, the magnitude of this effect was imprecisely estimated, was based on a heterogeneous and partly circular reference standard across studies, spanned a clinically and biologically diverse population, did not account for potential confounding by blood products or coagulative necrosis, and was derived from a search that did not include Embase or Scopus; these findings should therefore be regarded as preliminary and hypothesis-generating rather than as ready to inform clinical thresholds.

Time- and treatment-dependent performance further indicates that APTw MRI, if validated in prospective and more homogeneous cohorts, would need to be interpreted together with treatment timing, anti-angiogenic therapy, and conventional MRI findings, with likely greatest relevance in early post-radiotherapy evaluation.

## Figures and Tables

**Figure 1:**
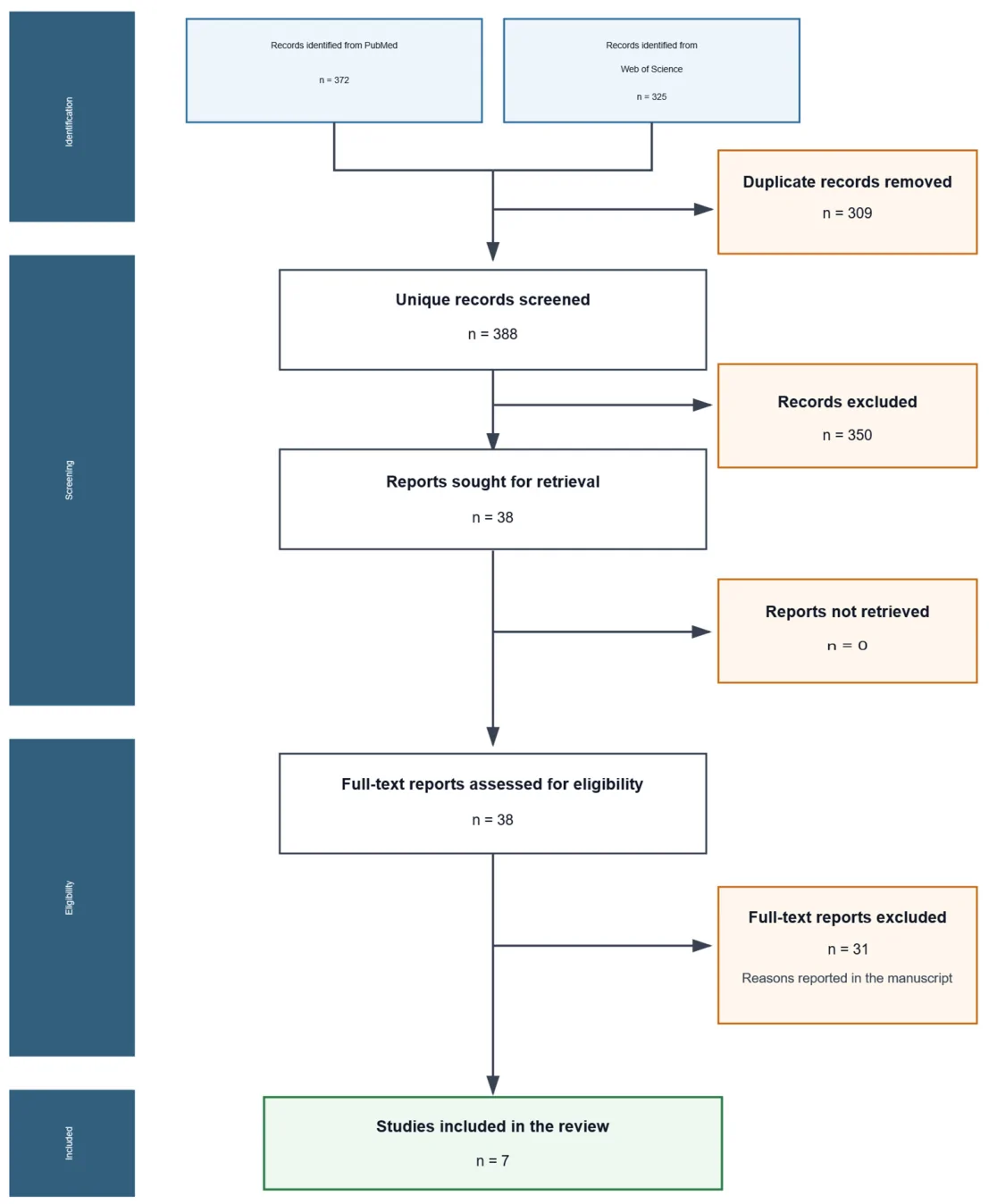
PRISMA 2020 flow diagram of the study selection process. The search identified 372 records from PubMed and 325 records from Web of Science. After removal of 309 duplicates, 388 unique records were screened, 38 full-text reports were assessed for eligibility, and seven studies were included.

**Figure 2:**
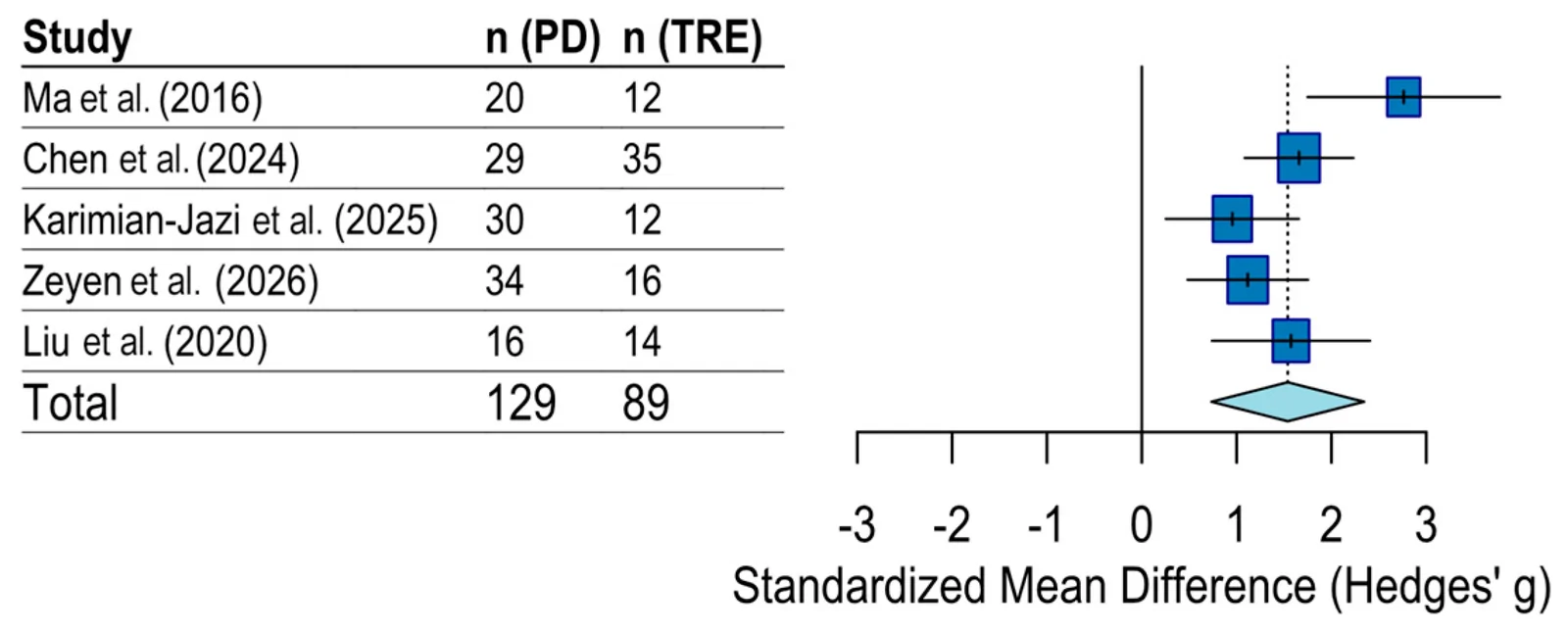
Forest plot of the standardized mean difference (Hedges’ *g*) in APTw signal between progressive disease (PD) and treatment-related effects (TRE) across the five studies with extractable continuous data by Ma et al. [14], Chen et al. [19], Karimian-Jazi et al. [20], Zeyen et al. [21], and Liu et al. [15] (random-effects, restricted maximum likelihood with Hartung–Knapp adjustment; pooled Hedges’ *g* = 1.54; 95% CI, 0.73–2.35; *p* = 0.006).

**Figure 3:**
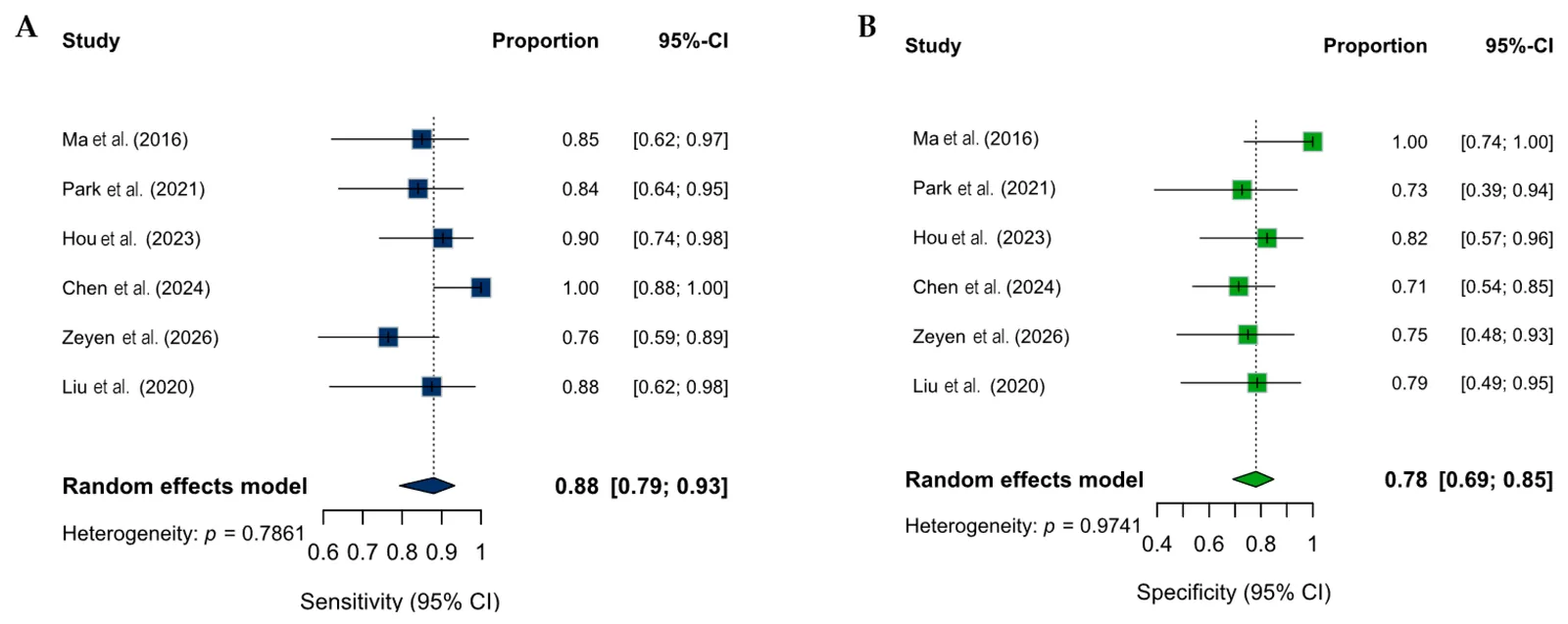
Coupled forest plots of APTw MRI in distinguishing progressive disease (PD) from treatment-related effects (TRE) (*k* = 6; random-effects model, logit transformation) across the included primary studies by Ma et al. [14], Park et al. [16], Hou et al. [17], Chen et al. [19], Zeyen et al. [21], and Liu et al. [15]. (**A**) Pooled sensitivity = 0.88 (95% CI, 0.79–0.93). (**B**) Pooled specificity = 0.78 (95% CI, 0.69–0.85).

**Figure 4:**
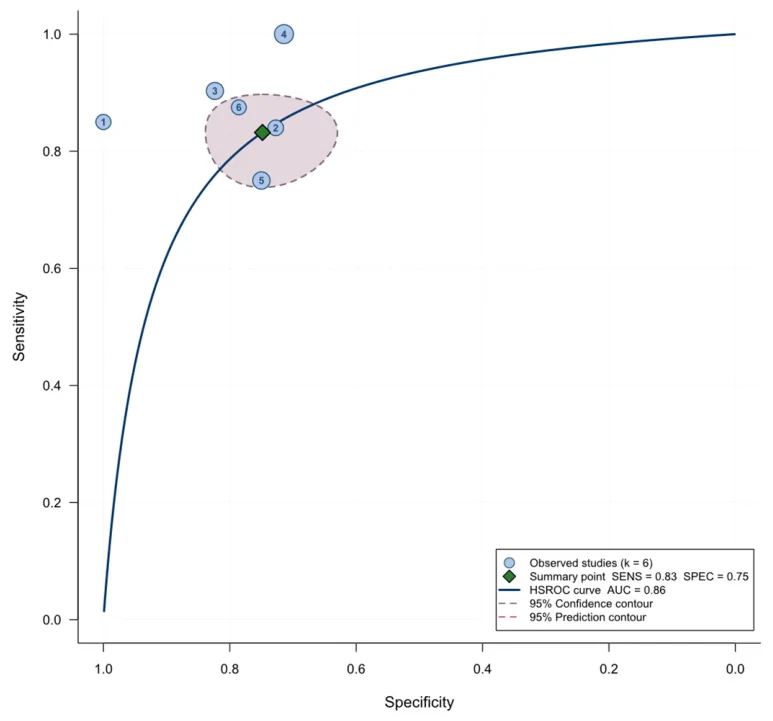
HSROC curve for APTw MRI in distinguishing PD from TRE (*k* = 6; bivariate Reitsma model; AUC = 0.86). Diamond indicates the summary operating point (sensitivity = 0.83, specificity = 0.75); dashed ellipses represent 95% confidence and prediction contours. Study identifiers: 1, Ma et al. [14]; 2, Park et al. [16]; 3, Hou et al. [17]; 4, Chen et al. [19]; 5, Zeyen et al. [21]; 6, Liu et al. [15].

**Figure 5:**
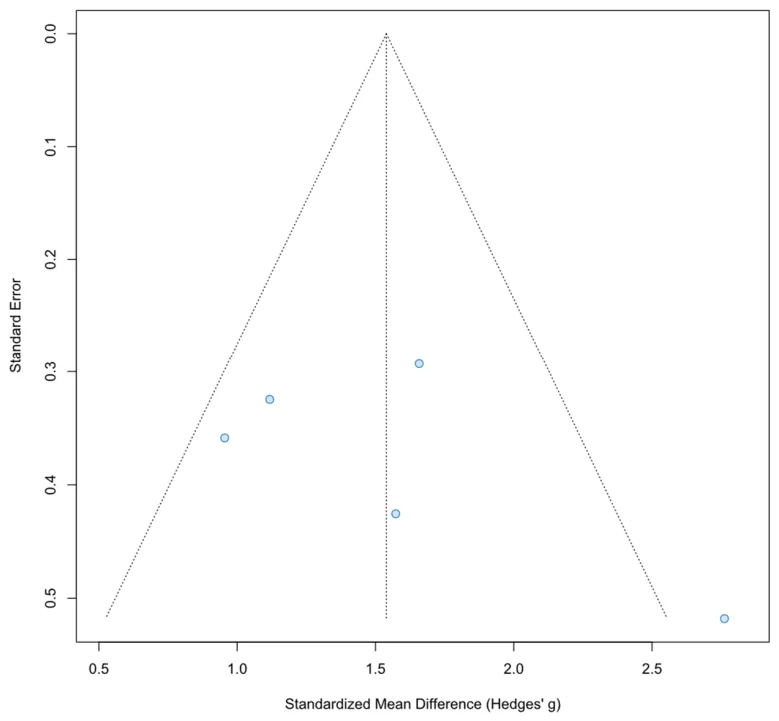
Funnel plot of SMD (Hedges’ *g*) across the five studies contributing to the primary analysis. The dashed vertical line indicates the pooled estimate (*g* = 1.54). Egger’s test was not performed because fewer than 10 studies were available; visual inspection suggests possible asymmetry, with Ma et al. (2016) [14] as an outlier.

**Figure 6:**
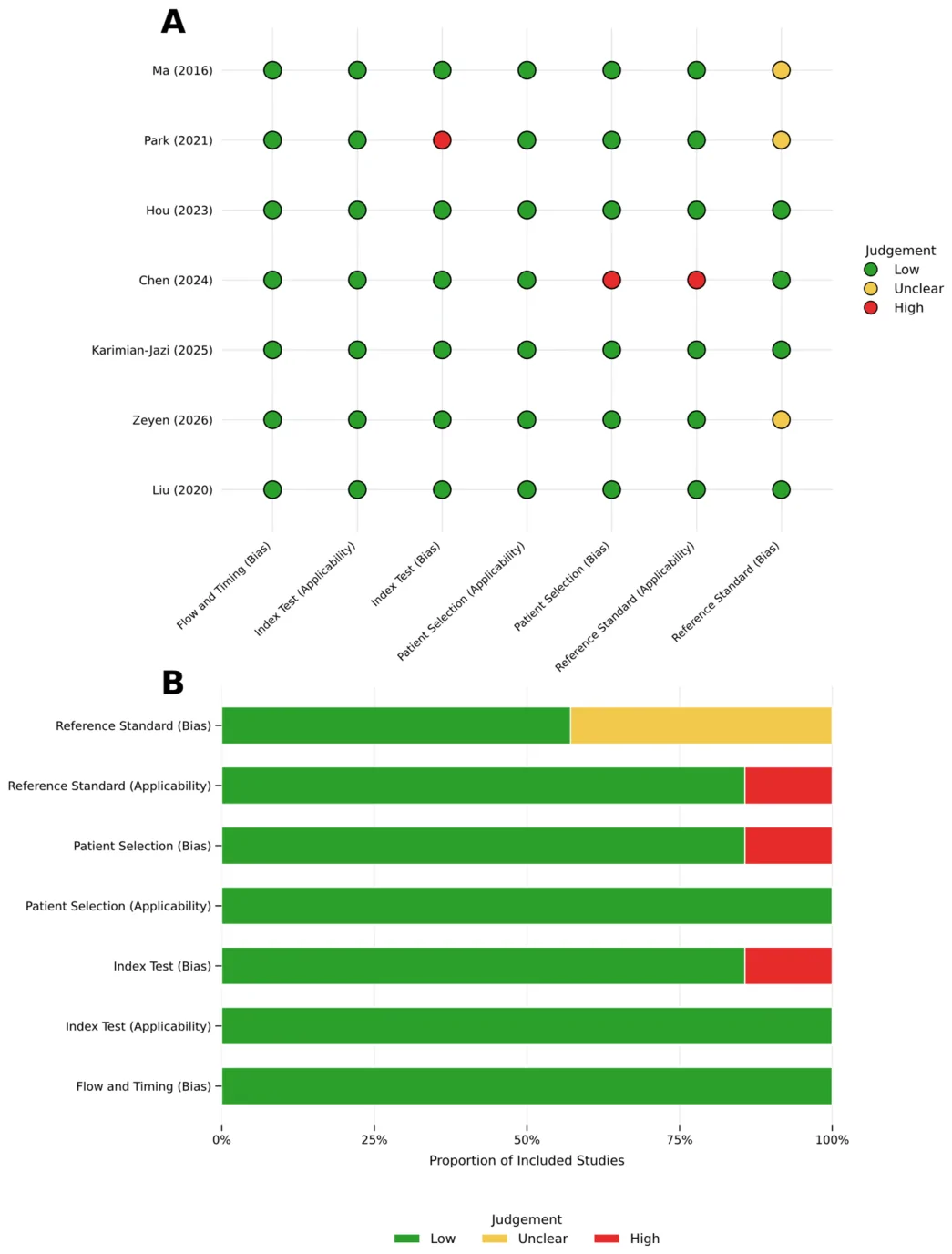
Quality assessment of included studies using the QUADAS-2 tool. (**A**) Methodological quality summary plot showing risk of bias and applicability concerns for each included study (Ma et al. [14], Park et al. [16], Hou et al. [17], Chen et al. [19], Karimian-Jazi et al. [20], Zeyen et al. [21], and Liu et al. [15]). (**B**) Methodological quality graph presenting overall proportion of studies with low, unclear, or high risk of bias and applicability concerns across domains.

**Table 1:** Characteristics of the Seven Included Studies.

Study	Year	WHO Grade	n (PD/TRE)	APTw Metric	Acq.	Reference Standard	Time RT to MRI, mo	Histo. Conf., %
Zeyen et al. [21]	2026	IV	34/16	Mean, voxel-based	3D	RANO 2.0 follow-up	11.3, median; IQR, 5.1–22.5	0
Karimian-Jazi et al. [20]	2025	II–IV	30/12	Mean, ROI-based	2D	RANO 2.0 + consensus	PD: 19; TRE: 10, mean	0
Chen et al. [19]	2024	III–IV	29/35	Mean, ROI-based	3D	Histopathology, 39%, + follow-up	NR	39
Hou et al. [17]	2023	III–IV	31/17	Mean, rAPTw	3D	Histopathology, 40%, + follow-up	PD: 8.8; TRE: 10.9, mean	40
Park et al. [16]	2021	II–IV	25/11	Mean, APT asymmetry	3D	Histopathology, 19%, + follow-up	PD: 18.1; TRE: 7.3, mean	19
Liu et al. [15]	2020	II–IV	16/14	Mean, ROI-based	2D	Histopathology, 23%, + follow-up	PD: 20.9; TRE: 27.4, mean	23
Ma et al. [14]	2016	III–IV	20/12	Mean, ROI-based	3D	Longitudinal follow-up, ≥6 mo	PD: 4; TRE: 2, median	0

Abbreviations: Acq., acquisition; APTw, amide proton transfer-weighted; Histo. conf., histopathological confirmation; IQR, interquartile range; mo, months; NR, not reported; PD, progressive disease; RANO, Response Assessment in Neuro-Oncology; rAPTw, relative APTw normalized to contralateral white matter; ROI, region of interest; RT, radiotherapy; TRE, treatment-related effects. Time RT to MRI indicates time from completion of radiotherapy to the APTw MRI scan used for analysis.

**Table 2:** Summary of Meta-Analysis Results.

Outcome	Estimate	95% CI	*p* Value	Heterogeneity/Notes
***Primary outcome (k = 5: Ma et al. [14], Chen et al. [19], Karimian-Jazi et al. [20], Zeyen et al. [21], Liu et al. [15]))***				
Pooled SMD (Hedges’ *g*)	1.54	0.73–2.35	0.006	Hartung–Knapp CI; I^2^ = 63.9% (95% CI, 0.0–96.7%), Q = 9.93 (df = 4, *p* = 0.04), τ^2^ = 0.235
95% prediction interval	0.04 to 3.04	—	—	Does not cross zero; reflects between-setting variability in the magnitude of the effect
***Sensitivity analysis (leave-one-out)***				
LOO SMD range	1.33–1.69	—	—	All estimates remained positive and clinically meaningful; omission of Ma et al. [14] yielded the lowest estimate (1.33) and omission of Karimian-Jazi et al. [20] the highest (1.69)
***Exploratory weighted mean APTw signal (k = 4: Ma et al. [14], Chen et al. [19], Karimian-Jazi et al. [20], Zeyen et al. [21])***				
Weighted mean APTw signal—PD	2.85%	—	—	SD = 0.40; inverse-variance weighted across positive-scale studies with extractable continuous data
Weighted mean APTw signal—TRE	1.75%	—	—	SD = 0.61; reported optimal cutoffs ranged from 1.97% to 2.75% (*k* = 3)
***Secondary outcomes—diagnostic test accuracy (k = 6: Ma et al. [14], Park et al. [16], Hou et al. [17], Chen et al. [19], Zeyen et al [21], Liu et al. [15])***				
Pooled sensitivity	0.88	0.79–0.93	—	Logit-transformed random-effects model
Pooled specificity	0.78	0.69–0.85	—	Logit-transformed random-effects model
HSROC AUC	0.86	—	—	Bivariate Reitsma model; summary point: sensitivity = 0.83, specificity = 0.75
***Publication bias***				
Egger’s test	Not performed	—	—	Fewer than 10 studies were available; funnel plot suggested possible asymmetry, with Ma et al. (2016) [14] as an outlier

Abbreviations: APTw, amide proton transfer-weighted; AUC, area under the curve; CI, confidence interval; df, degrees of freedom; HSROC, hierarchical summary receiver operating characteristic; *k*, number of studies; LOO, leave-one-out; PD, progressive disease; SD, standard deviation; SMD, standardized mean difference; TRE, treatment-related effects; τ^2^, between-study variance. The confidence interval for the pooled SMD is the Hartung–Knapp interval. The 95% prediction interval and the confidence interval for I2 are reported to convey the imprecision inherent to a small number of studies. Between-study heterogeneity for the diagnostic accuracy outcomes was negligible (sensitivity: I^2^ = 0%, *p* = 0.79; specificity: I^2^ = 0%, *p* = 0.97; Cochran Q test).

## Data Availability

The data extracted for this meta-analysis are available within the article and its Supplementary Materials (Table 1 and Table 2, Supplementary Table S1, PRISMA 2020 checklist).

## Supplementary Materials

The following supporting information can be downloaded at: https://www.mdpi.com/article/10.3390/life16101584/s1, Table S1: Representative studies excluded after full-text assessment [11,18,22,24,35,36,37,38,39].

## Abbreviations

APTw, amide proton transfer-weighted; AUC, area under the curve; CEST, chemical exchange saturation transfer; CI, confidence interval; df, degrees of freedom; FN, false negative; FO, false positive; HSROC, hierarchical summary receiver operating characteristic; IDH, isocitrate dehydrogenase; *k*, number of studies; LOO, leave-one-out; MGMT, O^6^-methylguanine-DNA methyltransferase; MRI, magnetic resonance imaging; MTRasym, magnetization transfer ratio asymmetry; PD, progressive disease; QUADAS-2, Quality Assessment of Diagnostic Accuracy Studies 2; RANO, Response Assessment in Neuro-Oncology; ROI, region of interest; SD, standard deviation; SMD, standardized mean difference; TN, true negative; TP, true positive; TRE, treatment-related effects; WHO, World Health Organization; τ^2^, between-study variance; 1p/19q, 1p/19q chromosomal codeletion.
