# Supplementary material for: Threshold-Independent Amide Proton Transfer-Weighted MRI in Post-Treatment Gliomas: A Systematic Review and Meta-Analysis of Effect Size and Diagnostic Accuracy
Source: Life (Basel). 2026 Sep 22;16(10):1584. doi: 10.3390/life16101584 (PMC13705705; doi:10.3390/life16101584)
Supplement: Supplementary file 1 [file life-16-01584-s001.zip › life-4519151-supplementary.pdf]

Supplemental Table S1: Representative Studies Excluded after Full-Text Assessment

Study	Reason for exclusion
Chen K et al. Front Oncol 2022 [22]	Systematic review/meta-analysis, not a primary study
Taylor C et al. Sci Rep 2022 [24]	Systematic review/meta-analysis, not a primary study
Guo P et al. NeuroImage Clin 2022 [35]	Data not extractable: deep-learning classification study reporting model performance rather than the APTw signal, with scan-level (non-independent) observations
Friismose AI et al. Radiography 2022 [36]	Data not extractable: correlational design without an extractable group-level APTw mean and standard deviation
Huang Q et al. Magn Reson Imaging 2023 [18]	ROI placed outside the contrast-enhancing region
Park KJ et al. Eur Radiol 2016 [37]	APT quantified as a 90th-percentile histogram parameter (APT90), not mean MTRasym(3.5 ppm), and therefore not comparable for the SMD analysis; stand-alone sensitivity and specificity for APTw not reported (only AUC and cross-validated multiparametric combinations)
Park JE et al. Eur Radiol 2018 [38]	Treatment-related change group n = 5; APT quantified as maximum/90th-percentile values (APTmax/APT90), not mean MTRasym(3.5 ppm); group mean ± SD for PD versus TRE not extractable
Chan RW et al. J Neurooncol 2021 [39]	Not performed at 3 Tesla
Zhou J et al. Nat Med 2011 [11]	Preclinical animal study (rat model); cited as biological ground truth in the Discussion only

Abbreviations: APT, amide proton transfer; APT90, 90th-percentile histogram value of APT; APTmax, maximum APT value; APTw, amide proton transfer-weighted; AUC, area under the curve; MTRasym, magnetization transfer ratio asymmetry; PD, progressive disease; ROI, region of interest; SD, standard deviation; SMD, standardized mean difference; TRE, treatment-related effects.

This table lists representative full-text exclusions. All 31 full-text exclusions and their reasons are summarized by category in the study-selection flow diagram (Figure 1).
